# Empirical Modeling of Seasonal Cooling Performance Based on Test Devices Using Zinc Oxide/Low-Density Polyethylene Passive Cooling Membranes

**DOI:** 10.3390/polym17101420

**Published:** 2025-05-21

**Authors:** Yinjia Zhang, Jun Natsuki, Chengwu Weng, Vuong Dinh Trung, Yiwen Wang, Lina Cui, Toshiaki Natsuki

**Affiliations:** 1 College of Textiles and Apparel, Quanzhou Normal University, Quanzhou 362000, China; 20hs154b@shinshu-u.ac.jp; 2 Interdisciplinary Graduate School of Science and Technology, Shinshu University, Ueda 386-8567, Nagano, Japan; 22hs153a@shinshu-u.ac.jp; 3 Key Laboratory of Clothing Materials of Universities in Fujian, Quanzhou Normal University, Quanzhou 362000, China; 4 Institute for Fiber Engineering and Science (IFES), Interdisciplinary Cluster for Cutting Edge Research (ICCER), Shinshu University, Ueda 386-8567, Nagano, Japan; jnatsu@shinshu-u.ac.jp; 5 Comprehensive Technology Service Center of Quanzhou Customs, Quanzhou 362300, China; weng_ecjtu@163.com; 6 College of Textile and Clothing, Xinjiang University, Urumqi 830046, China; 107552304806@stu.xju.edu.cn; 7 Faculty of Textile Science and Technology, Shinshu University, 3-15-1 Tokida, Ueda 386-8567, Nagano, Japan

**Keywords:** passive cooling, composites, thermal management, seasonal variation

## Abstract

Outdoor structures, such as vehicles, buildings, and outdoor equipment, are prone to overheat due to prolonged exposure to solar irradiation, which could affect their service life or user experience. To address this urgent issue, we developed a climate-adaptive thermal management solution using zinc oxide (ZnO)/low-density polyethylene (LDPE) hybrid membranes. The cooling performance of the membrane was examined across different seasons, achieving maximum temperature reductions (∆T) of 12.55 °C in summer, 8.02 °C in autumn, and 2.90 °C in winter. Our results demonstrated that the material’s cooling efficiency varied with seasonal solar irradiance, showing quicker responsiveness in summer and reduced in winter, effectively preventing overcooling. Moreover, the enclosed specific volume (*SV*) was identified as another critical parameter affecting cooling performance. We established an empirical correlation between ∆T and *SV* to quantify passive cooling performance across different seasons. This standardized method for assessing the cooling effect enables comparison between different materials, which is essential for determining climate-adaptive thermal management. Notably, the ZnO/LDPE membranes exhibited stable and balanced performance year-round, highlighting their potential for substantial energy savings in outdoor applications. This research provided valuable insights for designing climate-adaptive passive cooling materials that optimize thermal management across seasonal variations while contributing to sustainable energy conservation.

## 1. Introduction

The Earth’s climate has warmed more than 1.0 °C over the past 100 years [1]. The processes of global warming may be speeded as the population grows and cities expand [2,3,4]. As global warming and urban heat island effects intensify, cooling demands for vehicles and buildings continue to rise rapidly. Cooling is one of the major energy consumers globally and a primary driver of peak electricity demand, with air conditioning accounting for 15% of the total energy used in U.S. commercial buildings and 70% of the total electricity consumption in Saudi Arabia [5]. Glass, widely used for its excellent transparency, remains an essential component in building facades and vehicle windows. However, conventional glass transmits partial of solar irradiation, significantly increasing interior temperatures and, consequently, raising electricity consumption [6]. On the other hand, the essential resources for human survival (e.g., fossil fuels, fresh water, and arable land) are becoming increasingly scarce due to rapid population growth and unsustainable consumption patterns [7,8]. The depletion of these resources, coupled with the escalating energy demand for cooling systems, poses significant challenges to global sustainability. Hence, slowing down the speed of global warming and saving energy become strategies globally [9,10]. More and more researchers have been attracted to the field of developing passive cooling materials to counter the challenges, which have the function of cooling without any energy input [11,12,13,14,15,16]. The theoretical basis of passive cooling materials depends on their selective transmission of the short-wave range or high-energy partial of thermal radiation. These materials have two properties: lower solar transmittance (0.2–2.5 µm) that minimizes solar heat input [6] and stronger mid-infrared (MIR) transmittance in the atmosphere’s window (8.0–13.0 µm) that creates heat loss [17,18,19,20,21,22,23].

Current passive cooling materials used for vehicles and buildings mainly include reflective metal mirrors, coating, white structural wood, spectral selective materials, and composites. Numerous researchers, represented by Raman et al., have made significant contributions to this field, proving valuable guidance for future studies [24,25,26,27,28,29,30,31,32,33,34,35,36,37,38]. However, it is important to highlight that the evaluation standards for cooling effects have not been established across the reports. This variation is primarily manifested in the diversity of cooling performance testing equipment and different seasonal tests for evaluation (Table S1). Such inconsistencies have made it difficult to directly compare results across different studies and hinder the establishment of standardized assessment protocols. Different self-made devices presently include some that were open devices [37,39,40], while others were sealed but varied in the volumes of the air pocket, and the window areas covered by materials were also different [34,35,41,42]. The enclosed The enclosed volume-to-window area ratio, which we defined as specific volume (*SV*) in this work, involved in the passive cooling test was found to be critically important in Cui’s research [43]. The research indicated that the cooling effect declines exponentially as *SV* increases. Accordingly, to evaluate the cooling effects of passive cooling materials used in vehicles and buildings, testing should be conducted under their corresponding *SV* test conditions. On the other hand, seasonal performance variation remains a critical but often neglected aspect in the practical application of passive cooling materials. For outdoor structures, the key challenge in energy management is ensuring year-round energy efficiency rather than relying solely on short-term cooling during summer months. The effectiveness of passive cooling materials might fluctuate significantly throughout the year due to seasonal variation, incidence angles, and ambient temperatures. Current research predominantly evaluates performance under singular climatic conditions, typically emphasizing summer cooling efficiency, while comprehensive assessments of seasonal adaptability remain scarce. However, given global warming trends and the demands of certain specialized applications, it is equally important to consider cooling performance in seasons [39]. Furthermore, the field currently lacks standardized evaluation methods that would enable systematic comparison of materials’ cooling performance across different seasons.

Inspired by these considerations, here we first prepared a composite in which the zinc oxide (ZnO) nanoparticles (NPs) were uniformly dispersed in low-density polyethylene (LDPE) as a kind of passive cooling composite. Recognizing that the size of the test boxes significantly influenced the cooling measurements, we designed an innovative test device with an adjustable enclosed box volume and window area. This experimental setup enabled systematic evaluation of how the *SV* affects measured cooling performance across different seasons. We comprehensively evaluated the material’s cooling efficacy during summer, autumn, and winter, which led to the establishment of empirical correlations between cooling performance and the *SV* ratio. These seasonal empirical models would represent the standardized framework for objective cross-material comparison of passive cooling performance, addressing a critical gap where inconsistent test boxes have hindered meaningful comparisons between different studies. Our approach not only quantified how the *SV* of the test box influenced cooling measurements but also provided standardization that accounted for seasonal variations. The findings provided a theoretical and experimental foundation for developing next-generation passive cooling materials with stable year-round performance, ultimately enabling sustained optimization of energy consumption in buildings and vehicles while ensuring consistent indoor thermal comfort throughout all seasons.

## 2. Fabrications and Experimentation

### 2.1. Materials

ZnO NPs with an average size of 90 nm were obtained from Rhawn Co., Ltd., (Shanghai, China). LDPE pellets (AS: 9002-88-4, melting index of 25.0 g/10 min) were supplied by Alorich Chemistry Co., Inc., (Wood Dale, IL, USA). Xylene was obtained from Kanto Chemical Co. Inc., (Tokyo, Japan). All materials were used directly without any purification and treatment.

### 2.2. Preparation of ZnO/LDPE Membranes

Given the guidance from previous research, we experimentally fabricated the ZnO/LDPE membrane by 6% concentration of ZnO NPs, and 0.06 g of ZnO NPs, which were commercial products as a filter, were added to 20.0 g of xylene, used as a solvent by sonicating for 12 min and then mixed with 1.0 g of LDPE pellets. The mixture solutions were prepared by magnetic stirring under an oil bath at 115 °C for 300 h. The solution, which contained well-dispersed ZnO NPs, was evaporated in a vacuum oven at a temperature of 125 °C and hot pressed into 0.2 mm thick membranes. The membranes were labeled as ZnO/LDPE.

### 2.3. Characterization and Evaluation

The surface morphology, elemental mapping, and analysis were examined using a scanning electron microscope (SEM, Zeiss MERLIN, Oberkochen, Germany). The average particle size distributions of the ZnO NPs were computed using Image-Pro Plus software (Version 6.0.0.260) from SEM images. The thermal properties were tested with differential scanning calorimetry (DSC, Thermo plus EVO2 DSC vesta, Rigaku, Tokyo, Japan) at a heating/cooling rate of 10 °C/min in the temperature range from −80 °C to 300 °C in a nitrogen atmosphere. The membrane was tensile tested by a universal testing machine (Microtester 5948, Instron, Norwood, MA, USA) at a strain rate of 0.10 min^−1^ with a load cell of 100 N capacity under 25 °C and 60% relative humidity. Solar transmittance in the range of 0.2–2.5 µm was measured using an ultraviolet-visible-near-infrared (UV-Vis-NIR) spectrophotometer (AQ6375B, Yokogawa, Tokyo, Japan), while solar transmittance in the range of 2.5–17.0 µm was measured using Fourier transform infrared spectroscopy (FTIR, IR Prestige-21, Shimadzu, Tokyo, Japan).

### 2.4. Cooling Performance Test

The passive cooling performance of the membranes under different *SV*s and seasons were evaluated with a self-assembled passive cooling testing system. The test box was sealed, made of foam, and wrapped with aluminum foil to minimize the impacts of conductive and convective heat losses and environmental radiation. The standardized internal size of the box was 400 mm in length, 260 mm in width, and 190 mm in height. The internal sizes were adjustable, with the interior air pocket volume denoted as $V_{b}$. A circular window was at the top of the test box, with its area size designated as $A_{w}$. A temperature sensor was installed near the window to monitor the interior air temperature. In our previous research, we defined the ratio between the volume of the interior air pocket $V_{b}$ and the window area size $A_{w}$ to be the specific volume (*SV*) [43]:

(1) $$SV=\frac{V_{b}}{A_{w}}\;\left(m\right)$$

The *SV* of the test box served as an analog for the relationship between the window and interior space of vehicles and buildings. Our previous investigations have established that the *SV* values typically range around 0.05–4 m for vehicles and commercial offices with large windows, whereas residential buildings demonstrate that *SV* values could be about 9–23 m. To comprehensively evaluate this, the *SV*s of our test box ranges were designed to span 0.06–21 m.

### 2.5. Principles and Applications

**Figure 1:**
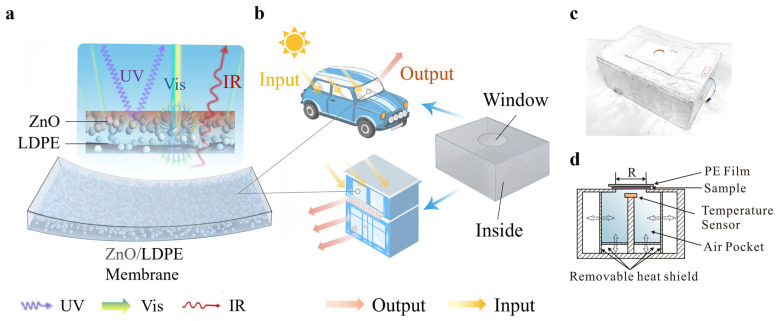
(**a**) Schematic of the ZnO/LDPE membrane. (**b**) Comparison between the test box and simulation of practical applications. (**c**) Photo of test box. (**d**) Cut-out schematic of the test box through the middle, showing how an air pocket was created.

Currently, an effective strategy for passive cooling is to reduce the transmittance of solar irradiance while efficiently transmitting infrared (IR) radiation. Recently proposed composites with uniformly distributed optical resonators could effectively achieve these optical properties. Specifically, a common approach is to use NPs in an appropriate size as resonators, while organic materials serve as the IR transmitting substrate. Based on these findings, we would design a passive cooling membrane composed of ZnO NPs incorporated into an LDPE matrix. As shown in Figure 1a, the ZnO/LDPE membrane architecture is intended to synergistically combine the optical functionalities of both materials. LDPE was selected as the matrix material due to its excellent IR transparency. This property stems from its simple chemical structure comprising only aliphatic C–C and C–H bonds, which exhibit minimal vibrational absorption in the MIR range. This allows thermal radiation emitted from heated surfaces to pass through the membrane with minimal loss, facilitating efficient cooling. In addition, LDPE offers desirable mechanical flexibility, chemical stability, low cost, and compatibility with large-area processing, making it suitable for scalable deployment. However, LDPE alone is not effective in blocking solar radiation due to its high transmittance across the solar spectrum (300–2500 nm), especially in the ultraviolet (UV) and visible (Vis) regions. To complement this property, ZnO NPs with a 90 nm diameter were incorporated as a filler. ZnO NPs was chosen because of its high refractive index and strong scattering capability in the UV–Vis range, which are well explained with the Mie scattering theory [44]. The combination of LDPE and ZnO provides an effective design. LDPE ensures efficient IR emission for passive cooling, and ZnO enhances solar shielding through optical scattering. Together, they form a hybrid membrane with great potential for practical applications in vehicle exteriors, building facades, and other outdoor infrastructure that requires sustainable thermal management.

The heat gains for vehicles or buildings exposed under solar irradiation was illustrated in Figure 1b. The total heat gains could be defined as:

(2) $$P_{heat\;gains}=P_{sun}+P_{envi\;rad}-P_{cond}-P_{cabin\;rad}-P_{conv}$$

where $P_{sun}$ and $P_{envi\;rad}$ is, respectively, the heat gain rate from solar irradiance and environmental radiation, shown as input. $P_{cond}$, $P_{cabin\;rad}$, and $P_{conv}$ are the net heat loss rates through conduction, radiation from inside of the cabin, and convection, respectively, shown as output.

Maintaining cabin thermal comfort, therefore, involves the minimization of heat gains by reducing outsider heat gain and enhancing heat loss [45,46]. The glass of vehicles or buildings exhibit a transmittance exceeding 85% in solar irradiance (AM 1.5) (0.4–2.5 µm) [47], while being almost completely opaque in the atmosphere’s window. This optical characteristic facilitates the transmission of high-energy photons from solar irradiation while blocking the escape of MIR radiation. Consequently, extended exposure to solar irradiation might cause temperatures to progressively rise inside enclosed vehicles or buildings. This thermal accumulation effect could be experimentally replicated using a sealed test box in our self-assembled passive cooling performance evaluation system. Furthermore, for vehicles and buildings where heat accumulates internally, heat exchange through windows could affect cooling energy consumption. In our test, the transparent window and inside of the test box simulated as the window and cabin of vehicles or buildings, respectively. Figure 1c presented the photograph of the test box. Figure 1d showed a cross-section view of the test box. The test sample was lined over the window and covered with a polyethylene (PE) film to eliminate the influence of air convection [48]. It is worth noting that operating vehicles and buildings would generate heat from internal sources such as engines or indoor equipment. While these internal heat sources would increase the overall cooling demand, our study focused specifically on characterizing the passive cooling membrane’s performance under solar irradiation.

Furthermore, seasonal fluctuations in solar irradiation resulted in variable thermal gains, which inherently affect cooling performance. Hence, we further considered incorporating seasonal variation as an additional experimental variable to evaluate the cooling performance of the passive cooling composites across different seasonal conditions. Due to minimal differences in weather conditions between spring and autumn at the experimental site, only summer, autumn, and winter were selected as representative seasons for the annual cycle. The experimental investigation assessed the temperature reduction capabilities of the samples under varying *SV* test configurations across the three selected seasonal periods.

## 3. Results and Discussion

### 3.1. Characterization of the ZnO/LDPE Membrane

**Figure 2:**
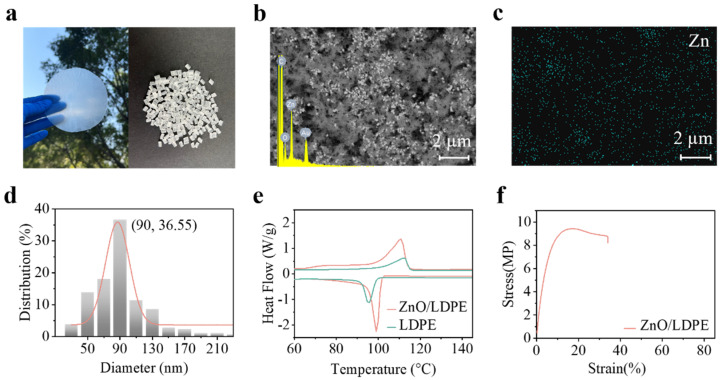
(**a**) The ZnO/LDPE membrane and master batches. (**b**) The surface morphology and elemental analysis. (**c**) EDX mapping of Zinc element. (**d**) ZnO NPs diameter distributions. (**e**) The DSC curves of the LDPE and ZnO/LDPE membrane. (**f**) The stress–strain curves for ZnO/LDPE membrane.

Figure 2a showed the morphology of ZnO/LDPE membrane and master batches, which exhibited transparency, making it suitable for application as a functional film to be adhered to windows of vehicles and buildings. Figure 2b showed the surface morphology and corresponding elemental analysis (insert) of the sample. The elemental analyses showed that membrane contained carbon, oxygen, and zinc, which could be from the raw materials of LDPE and ZnO NPs. The image of SEM illustrated that NPs were uniformly dispersed in the LDPE, while the EDX mapping of zinc (Figure 2c) confirmed this well dispersion. In addition, the ZnO NPs in the sample ranged from 50 to 210 nm in diameter, with 90 nm being the most predominant size as shown in Figure 2d. The dispersion of the NPs played a key role in designing a hybrid material for passive cooling. Since passive cooling relies fundamentally on optical properties, the dispersion of NPs would directly determine the materials’ performance. When particle sizes are comparable to the wavelengths of solar light, Mie scattering becomes strong [48]. Uniform dispersion of these NPs serves a dual purpose by optimizing individual scattering events while simultaneously enabling multiple scattering effects throughout the material. As light undergoes multiple scattering events, its propagation path lengthens significantly, increasing the probability that solar radiation will be reflected rather than absorbed. In the previous work, we identified that this enhanced scattering mechanism ultimately maximizes the material’s solar shielding [49]. This uniform dispersion satisfied our design guidelines.

The thermal properties of pure LDPE and ZnO/LDPE membrane were shown in Figure 2e. The crystallinity of pure LDPE films was 3.2%, while it was 37.9% for ZnO/LDPE samples. The incorporation of uniformly dispersed ZnO NPs provided better specific surface area and additional nucleation sites, effectively promoting crystallization. Theoretically, enhanced crystallinity could influence the light propagation pathway [50]. As a membrane, its mechanical property is a key indicator. The mechanical property of the membrane was shown in Figure 2f. The mechanical property of the membrane demonstrated exceptional strength but relatively limited ductility, which was fundamentally associated with the evolution of failure mechanisms from localized plastic deformation to defect-controlled fracture.

### 3.2. Optical Properties and Passive Cooling Performances

**Figure 3:**
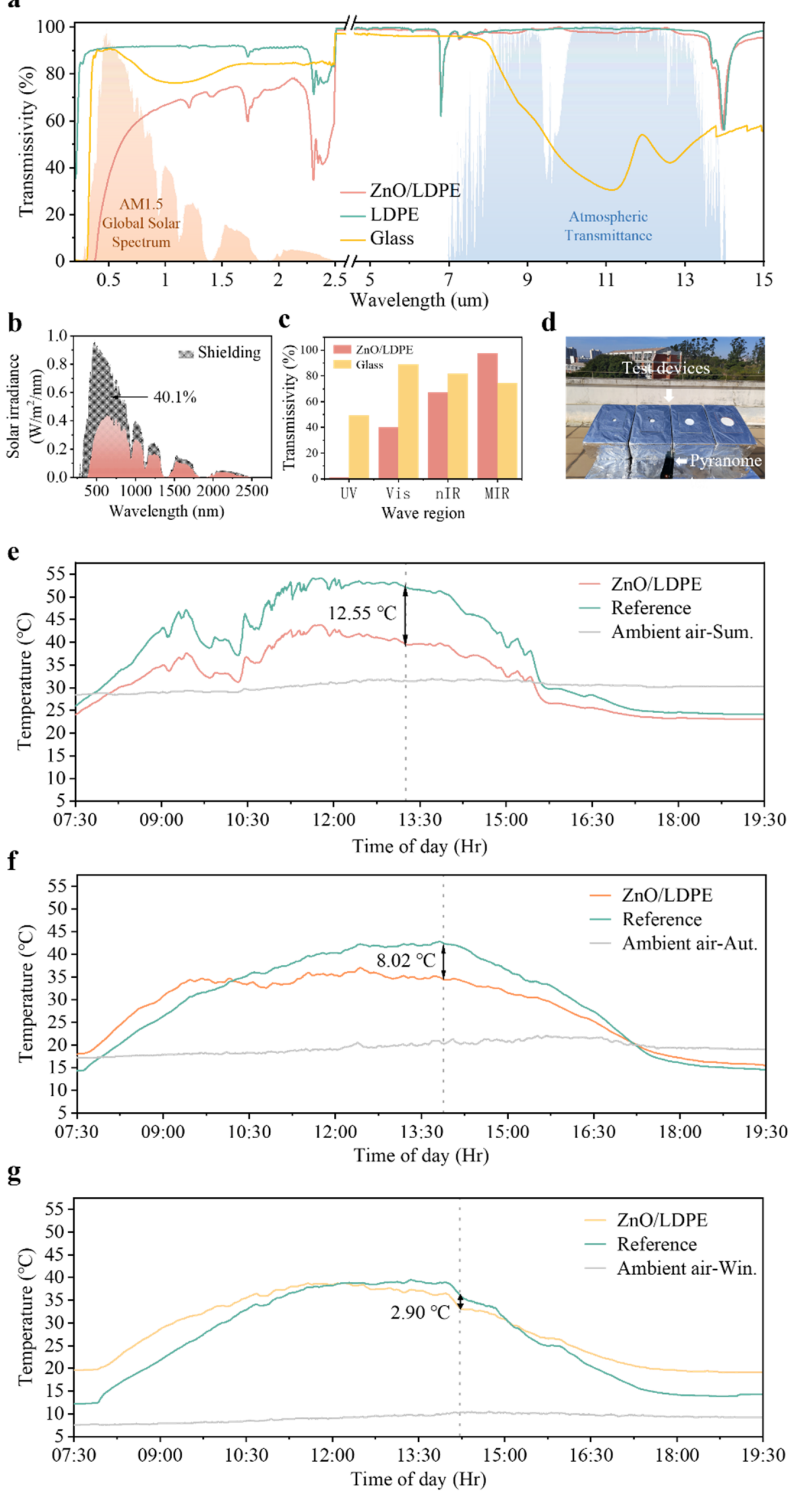
(**a**) Transmissivity spectra of ZnO/LDPE, pure LDPE, and glass from ultraviolet to mid-infrared range. The shaded areas showed the AM 1.5 solar spectrum (orange) and atmospheric spectrum (blue) for reference. (**b**) Shielding part of ZnO/LDPE in solar spectrum (AM 1.5). (**c**) The transmissivities of ZnO/LDPE membrane and glass in UV, Vis, nIR, and MIR regions, respectively. (**d**) A photograph of the experimental setup for testing passive cooling performance outside. Comparative cooling temperatures curves of the sample and LDPE (*SV* = 1.29) during (**e**) summer, (**f**) autumn, and (**g**) winter.

In theory, the optical performance of passive cooling materials requires low transmittance (or high reflectance) in the solar region and high transmittance (or high emittance) in the atmospheric window of the IR region. Our cooling membrane was designed for outdoor structures prone to internal heat accumulation. It functions by facilitating heat transfer from interior spaces to ultracold outer spaces, while minimizing solar heat absorption. Consequently, we focused on optimizing the material’s transmittance properties across relevant wavelengths. Figure 3a showed the transmissivities of ZnO/LDPE, LDPE, and glass, respectively. Glass demonstrated high transmittance in the solar spectral region due to its amorphous SiO_2_ network structure having minimal absorption in the UV–Vis–nIR range, and it exhibited significantly lower transmittance in the atmospheric window owing to the strong Si–O–Si vibrational absorption bands in this spectral region. This optical asymmetry leads to a greenhouse effect as solar irradiation readily passes through glass, but the resulting IR radiation from heat buildup struggles to escape, particularly under prolonged solar exposure. In contrast, ZnO/LDPE showed lower solar transmittance, while maintaining higher transmittance in the atmospheric window. This validated our strategic selection of proper hybrid materials for the realization of optical selection to effectively guarantee passive cooling. In the range of 7–14 µm, the ZnO/LDPE membrane retained an excellent optical property of LDPE. Figure 3b presented the solar irradiation shielding ratio of the samples under AM1.5, where the sample achieved an average shielding ratio of 40.1%. The transmissivities of the hybrid membrane and glass in different spectral regions were further compared in Figure 3c. The membrane exhibited negligible transmissivity of 1.0% in the UV region (200–400 nm), while glass was 49.6%. In the high energy portion of solar irradiance, in the Vis spectrum (400–700 nm), the transmissivity of the sample was 40.1%, while the glass was high at 89.0%. This solar block was due to Mie scattering by ZnO NPs, uniformly dispersed in a cross-matrix. This results further confirmed that our sample demonstrated superior solar irradiance resistance compared to traditional glass materials. In addition, the sample exhibited high transmissivity with an average of 97.6% in the MIR region attributed to the weak C–H and C–C vibrational modes of LDPE that weakly absorb in the 8–13 µm range, while glass was 74.5%. These optical properties synergistically enabled the dual functions of solar irradiation shielding and efficient heat dissipation required for passive cooling materials, where ZnO NPs provided solar scattering, while the LDPE matrix ensured IR transparency through the atmospheric window.

Outdoor daytime experiments were conducted to evaluate the thermal performance of the ZnO/LDPE membrane. The tests were carried out on the rooftop of a building located in Quanzhou, China (24°52′32.0″ N, 118°40′20.5″ E) for 12 h (Figure 3d). To evaluate the thermal regulation capability of the samples, experimental investigations were conducted separately during three seasons (summer, autumn, and winter). This multi-seasonal investigation systematically characterized the membrane’s cooling performance under varying ambient temperature conditions, thereby elucidating its thermal adaptive properties. The sample was covered on the window of the test box and pure LDPE film on another box as the reference for the direct sunlight experiments. Figure 3e–g presented the cooling temperature curves of the samples at test conditions with an *SV* value of 1.29 in summer, autumn, and winter, respectively. The *SV* value is in a common range of most vehicles and large window buildings. In our experiments, the ZnO/LDPE membrane achieved a maximum cooling temperature of 12.55 °C at 13:15 in summer, 8.02 °C at 13:53 in autumn, and 2.90 °C at 14:13 in winter. These results confirmed that the ZnO/LDPE membrane demonstrated a predominant cooling effect compared to the reference. ZnO/LDPE membranes consistently outperformed LDPE in cooling efficiency during summer. Interestingly, during autumn and winter, the internal temperatures of the control were lower than the ZnO/LDPE membrane during both the early experimental stages and before sunset. This could be attributed to the infrared transmission effect of LDPE outweighing the solar irradiation on the internal during these periods.

### 3.3. Mechanism of Seasonal Cooling Performance

**Figure 4:**
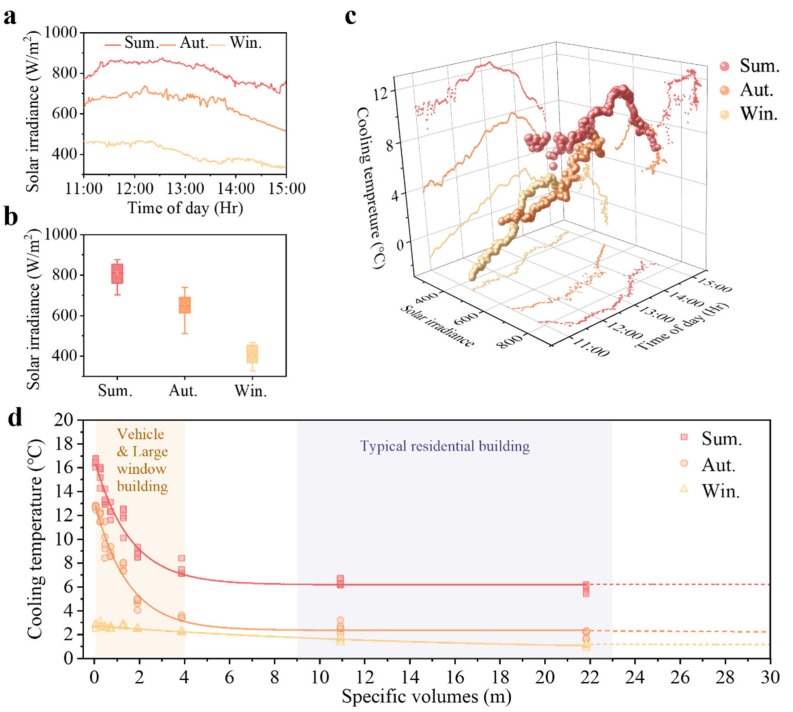
(**a**) Solar irradiation from 11:00 to 15:00 in summer, autumn, and winter, respectively. (**b**) Average solar irradiances in summer, autumn, and winter, respectively. (**c**) Three-dimensional relationship between cooling temperature, solar irradiance, and time of daytime across seasonal variations. (**d**) Temperature reduction as a function of *SV* in summer, autumn, and winter, respectively.

Seasonal changes in solar irradiance may have an influence. To further elucidate the seasonal variation in the cooling effect of the membrane, we conducted a comprehensive analysis of solar irradiance throughout the experiments. Figure 4a showed the solar irradiance variations recorded between 11:00 and 15:00 during the seasonal cooling performance tests under clear sky conditions. We chose this period as the sun nears its zenith, providing peak irradiance that represents the most challenging daytime thermal conditions. The irradiance peaked after 12:00 across all three seasons, reaching a maximum of 876.57 W/m^2^ in summer, 738.44 W/m^2^ in autumn, and 467.68 W/m^2^ in winter. These seasonal variations might arise from solar elevation changes, with summer’s higher altitude shortening the atmospheric path and reducing energy losses, while winter exhibits the opposite pattern. Autumn measurements showed greater fluctuation between 12:00 and 14:00, followed by a weakening. This instability likely reflects autumn’s characteristic atmospheric variability, including fluctuating cloud cover and temperature gradients. Specifically, the median solar irradiance values were 810.92 W/m^2^ for summer, 650.15 W/m^2^ for autumn, and 406.07 W/m^2^ for winter (Figure 4b). Summer and winter measurements exhibited minor variations, while autumn values fluctuate more, occasionally approaching either summer or winter levels.

Figure 4c showed a 3D plot of cooling temperature (∆T) versus solar irradiances (I) and time of day for three seasons. The cooling performance exhibited a clear seasonal pattern, with summer demonstrating the highest efficacy, followed by autumn, while winter showed the lowest values. This corresponded to the seasonal variations in solar irradiance, showing that the material’s cooling performance related to solar irradiance across seasons. The irradiance variations directly affect the thermal load on passive cooling materials, thereby determining their cooling performance across different seasons. The membranes worked best as a cooling candidate when sunlight was strongest. Notably, as depicted in Figure 4c, the ∆T curve in summer displayed more notable fluctuations than the corresponding irradiance curve, indicating enhanced sensitivity to solar irradiance variations during summer. This differential seasonal response suggested that the material’s varying sensitivity to solar irradiance changes, with the most pronounced and immediate response occurring during summer. Such behavior underscored the material’s adaptive cooling capabilities that were particularly advantageous during higher solar intensity. In Section 3.2, we observed that, during periods of low solar irradiance in the early morning and evening hours of autumn and winter, the experimental sample exhibited lower ∆T compared to the reference. This confirmed that the membranes exhibited varying sensitivity to solar irradiance across seasons. Under lower solar irradiance, it could avoid excessive cooling, which might contribute to energy efficiency. In contrast, LDPE demonstrated relatively limited cooling function in response to changing solar irradiances.

It was noteworthy that the experimental data revealed no positive correlation between ∆T and I. In fact, the solar irradiance at a single time point could not immediately affect the cooling performance. There existed a certain lag between peak cooling and peak irradiance, with the material’s maximum cooling effect occurring later than the peak solar irradiation time across all three seasons. This could be attributed to the amplitude attenuation and time delay of heat waves generated by external solar radiation as they were transmitted to the enclosed space, causing a time lag in the internal temperature rise, thereby affecting the material’s temperature regulation sensitivity. This thermal lag could be explained by Fourier’s heat conduction law [51] and thermal time constant theory [52,53]. The thermal response rates demonstrated seasonal variations. In summer, the intense sunlight accelerates heat radiation, shortening the lag time, while in winter, it decelerates, and in autumn it presents an intermediate state. During the experiments, the observed lag times varied seasonally, with approximately 45 min in summer, 2 h in autumn, and 2.5 h in winter. This further indicated the material’s higher temperature regulation sensitivity in summer (Table S2).

Certainly, this complex thermal regulation might also be influenced by factors such as environmental temperature differences, solar angle, latitude, or cabin dimensions [54,55,56]. Since our tests were conducted in a sealed chamber, eliminating the effects of ambient heat transfer and convection, we could characterize the membrane’s performance by considering only the solar irradiation effects. The *SV* of cabins might be another important influencing factor. According to the principles of energy conservation [57] and the Stefan–Boltzmann law [58], a smaller *SV* implies more heat dissipation surface per unit volume, allowing IR radiation to be transmitted outside more rapidly. Figure 4d showed the cooling curves of the sample corresponding to different *SV* values across three seasons. Overall, the sample exhibited the highest ∆T in summer and the lowest in winter. During summer and autumn, within the *SV* range of 0–4 m, ∆T sharply decreased as *SV* increased, stabilizing when *SV* larger than 4 m. Conversely, the ∆T curve in winter was like a line. Our previous research established that the *SV* significantly influenced the ∆T, with this relationship expressed as Equation (3) [43]:

(3) $$∆T=∆T_{\infty }+\left(∆T_{0}-∆T_{\infty }\right)\exp\left(-\frac{SV}{D}\right)$$

$∆T_{0}$ is the ∆T when *SV* → 0, which could be considered as the intrinsic cooling performance of the sample. $∆T_{\infty }$ is the ∆T when *SV* → ∞, as the lower bound of the cooling temperature. *D* represents the rate at which ∆T decreases as *SV* increases, which we refer to as the cooling effect diminishing parameter. A higher *D* value indicates a slower decline in ∆T with increasing *SV*.

Based on the tested data (Table S3), we analyzed the regression model that was carried out, and the summer model is shown in Equation (4) (R^2^ = 0.96):

(4) $$∆T_{Sum.}=6.02+10.63\exp\left(-SV/1.63\right)$$

The autumn model is shown in Equation (5) (R^2^ = 0.96):

(5) $$∆T_{Aut.}=1.60+11.43\exp\left(-SV/1.73\right)$$

The winter model is shown in Equation (6) (R^2^ = 0.93):

(6) $$∆T_{Win.}=1.04+1.72\exp\left(-SV/9.31\right)$$

The models effectively characterized the cooling performance of the membrane under varying *SV* values across different seasons. When *SV* → 0, ∆T reached its maximum theoretical values of 16.65 °C, 13.03 °C, and 2.76 °C for summer, autumn, and winter, respectively. These values represented the intrinsic maximum cooling capacity of the membrane. Conversely, as *SV* → ∞, ∆T converged toward lower limit values, reaching saturation points of 6.02 °C in summer, 1.60 °C in autumn, 1.04 °C in winter. These saturation values represented how much the membrane could passively reduce the temperature in open spaces across different seasons. It should be noted that these empirical models (Equations (4)–(6)) were developed based on experimental data collected in Quanzhou, China, with ambient temperatures ranging from 27 to 35 °C in summer, 18 to 23 °C in autumn, and 8 to 12 °C in winter. The validity of these correlations is, therefore, limited to similar climatic conditions and temperature ranges. For regions or years with different temperatures, the model parameters may require recalibration. The cooling ability exhibited seasonal dependence, with high saturation observed during summer and lower during autumn and winter. This corresponded with the Stefan–Boltzmann law [58], whereby the higher ambient temperatures in summer facilitate greater potential temperature gradients between the material and surrounding environment. Such performance aligned with the material’s optimized cooling properties under higher solar irradiance as observed in our experiments. Therefore, we hypothesized that ∆T correlates with seasonal variations in daytime solar irradiance. The solar irradiance was at 800 W/m^2^, 500 W/m^2^, and 400 W/m^2^ in summer, autumn, and winter, respectively. The cooling performance of the material appears to be governed by the intensity of incident solar irradiation. As a result, ∆T reached its maximum in summer. Autumn exhibits significant variability in solar irradiance, with minimum values approximating those of winter, thus explaining the comparable ∆T measurements observed between these seasons.

$∆T_{0}-∆T_{\infty }$ represents the range between maximum cooling potential and cooling saturation values, which correlates with the cumulative effects of seasonal solar irradiance. Theoretical analysis of comparative seasonal irradiation intensities suggested summer should exhibit maximum variation and winter minimum variation. However, empirical observations indicated that autumn experiences substantial fluctuations in solar irradiance, with certain periods exhibiting higher intensities than their corresponding temporal counterparts in summer. As a result, the cumulative effect of autumnal solar irradiation marginally exceeded that of summer, yielding enhanced cooling performance variation during autumn and diminished variability during winter.

The values of D showed seasonal variations, with summer having the smallest value, and winter having the largest. This suggested that the ∆T decline rate was more rapid during summer while slower in winter. These differences could likely be attributed to seasonal variations in solar irradiance and solar angle [59,60]. During winter periods, characterized by lower or insufficient solar irradiance input, the material exhibited a diminished rate of ∆T reduction with increasing *SV*. In contrast, summer and autumn experience relatively higher and comparable solar irradiation inputs, resulting in similar D values for these seasons, with autumn demonstrating marginally greater values than summer.

During summer and autumn, enclosed spaces like vehicles and buildings with large windows (*SV* = 0–4 m) experienced significant cooling from our membrane. This meant that cooling membranes enveloped to this type of space with a smaller *SV*, which tended to obviously cool down and could lead to substantial energy-saving potential. Conversely, as *SV* values increased (indicating more open spaces or smaller windows), the ∆T gradually decreased until reaching thermal equilibrium without further cooling. Overall, the membranes consistently demonstrated thermal regulation benefits across all seasonal variations, though with quantifiably different magnitudes.

## 4. Conclusions

In this study, the fabricated ZnO/LDPE membranes demonstrated excellent optical properties, blocking UV light, partially shielding Vis light, and transmitting most MIR radiation. Seasonal field tests revealed a strong correlation between solar irradiance and cooling performance. Maximum temperature reductions were 12.55 °C in summer, 8.02 °C in autumn, and 2.90 °C in winter. During summer, when sunlight is most intensive, the membrane showed stronger cooling effects and responded more sensitively to temperature changes. In the lower solar irradiance seasons like autumn and winter, the membrane had relatively lower cooling. This is an advantageous characteristic that contributes to its favorable performance across all seasons. We established a seasonal empirical relationship between *SV*s and their resultant cooling effects, introducing the standardized metric for the objective comparison of different materials. The development of such quantitative evaluation frameworks filled a critical gap in passive cooling material assessment. Our membranes exhibited stable performance in diverse applications, from buildings to vehicles, highlighting their versatility and year-round energy-saving potential. This comprehensive seasonal characterization opens pathways for future development of climate-specific formulations, integration with smart building systems, and the establishment of international testing standards. Future work should focus on expanding the empirical models to incorporate humidity and geographical locations. Additionally, research should consider the thermal effects from internal heat sources such as engines in vehicles or equipment in buildings during real-world applications to provide more comprehensive thermal management solutions. This work provides a valuable foundation for designing climate-adaptive thermal management systems for sustainable infrastructure, while the standardized evaluation methodology paves the way for accelerated commercialization of passive cooling technologies.

## Data Availability

The original contributions presented in this study are included in the article/Supplementary Materials. Further inquiries can be directed to the corresponding authors.

## Supplementary Materials

The following supporting information can be downloaded at: https://www.mdpi.com/article/10.3390/polym17101420/s1, Table S1: Comparison of the specific volume in different test devices and seasons for cooling measurement research; Table S2:Seasonal variation in timing of maximum temperature reduction (*ΔTmax*) and peak solar irradiance (*Imax*) for samples in outdoor conditions; Table S3: Cooling performance data of samples with different *SV* values across three seasons. Reference [61] are cited in the supplementary materials.
