# Supplementary material for: Empirical Modeling of Seasonal Cooling Performance Based on Test Devices Using Zinc Oxide/Low-Density Polyethylene Passive Cooling Membranes
Source: Polymers (Basel). 2025 May 21;17(10):1420. doi: 10.3390/polym17101420 (PMC12115205; doi:10.3390/polym17101420)
Supplement: Supplementary file 1 [file polymers-17-01420-s001.zip › polymers-3646256-supplementary.pdf]

## Supplementary Information

# Zinc oxide/low density polyethylene hybrid membranes for seasonal daytime passive cooling

**Yinjia Zhang [1,2,3], Jun Natsuki [4], Chengwu Weng [5], Vuong Dinh Trung [2], Yiwen Wang [6], Lina Cui [1,3,*]
and Toshiaki Natsuki [1,4,7,*]**

[1] College of Textiles and Apparel, Quanzhou Normal University, Quanzhou 362000, China; 20hs154b@shinshu-u.ac.jp
[2] Interdisciplinary Graduate School of Science and Technology, Shinshu University, Ueda 386-8567, Nagano, Japan; 22hs153a@shinshu-u.ac.jp
[3] Key Laboratory of Clothing Materials of Universities in Fujian, Quanzhou Normal University, Quanzhou 362000, China
[4] Institute for Fiber Engineering and Science (IFES), Interdisciplinary Cluster for Cutting Edge Research
(ICCER), Shinshu University, Ueda 386-8567, Nagano, Japan; jnatsu@shinshu-u.ac.jp
[5] Comprehensive Technology Service Center of Quanzhou Customs, Quanzhou 362300, China; weng_ecjtu@163.com
[6] College of Textile and Clothing, Xinjiang University, Urumqi 830046, China; 107552304806@stu.xju.edu.cn
[7] Faculty of Textile Science and Technology, Shinshu University, 3-15-1 Tokida, Ueda 386-8567, Nagano, Japan
* Correspondence: cuilina@qztc.edu.cn (L.C.); natsuki@shinshu-u.ac.jp (T.N.); Tel.: +86-0595-22900251 (L.C.); 81-268-21-5421 (T.N.); Fax: +81-268-21-5482 (T.N.)

**Table S1.** Comparison of the specific volume in different test devices and seasons for cooling measurement research.

| Research | Season | Specific volume (SV) (m) | Cooling performance (℃) |
|---|---|---|---|
| Sanghyun Jeon et al. [1] | Summer | +∞ | 7.4 |
| Aaswath P. Raman et al.[2] | Winter | +∞ | 4~5 |
| Dasol Lee et al.[3] | Spring | +∞ | 6.1 |
| S. Zeng et al. et al.[4] | Winter | Unspecified (estimated at 0.05) | 10.2 |
| Tian Li et al.[5] | - | estimate at 0.1 | 4.0 |
| Kamlesh Panwar et al.[6] | - | estimate at 0.33 | 3.0 |
| Hongkai Zhang et al.[7] | Summer | 0.15 | 5.6 |
| Song, Yingnan et al. [8] | - | - | 13.5 |
| Yijun Chen et al [9] | Summer | estimate at 1 | 15.6 |
| Wang Tong et al [10] | Spring, Winter | - | 8.9/5.5 |

**Table S2.** Seasonal variation in timing of maximum temperature reduction ($\Delta T_{max}$) and peak solar irradiance ($I_{max}$) for samples in outdoor conditions.

| **Season** | **Time of $\Delta T_{max}$** | **Time of maximum $I_{max}$** |
|---|---|---|
| Summer | 13:15 | 12:30 |
| Autumn | 13:53 | 12:12 |
| Winter | 14:13 | 12:07 |

**Table S3.** Cooling performance data of samples with different SV values across three seasons.

| Specific volume (SV) (m) | Cooling temperature (ΔT) in Summer (℃) | Cooling temperature (ΔT) in Autumn (℃) | Cooling temperature (ΔT) in Winter (℃) |
|---|---|---|---|
| | 16.01 | 12.77 | 2.77 |
| | 16.78 | 12.64 | 2.86 |
| 0.06 | 16.67 | 12.83 | 2.73 |
| | 16.45 | 12.46 | 2.46 |
| | 16.47 | 12.73 | 2.81 |
| | 15.86 | 12.12 | 2.62 |
| | 16.03 | 12.29 | 3.09 |
| 0.26 | 15.86 | 12.12 | 2.62 |
| | 15.16 | 11.42 | 2.72 |
| | 14.26 | 11.52 | 2.59 |
| | 12.94 | 9.23 | 2.52 |
| | 12.91 | 10.16 | 2.56 |
| 0.47 | 14.2 | 11.46 | 2.57 |
| | 13.15 | 8.41 | 2.53 |
| | 13.32 | 9.56 | 2.66 |
| | 12.28 | 8.54 | 2.69 |
| | 12.29 | 8.55 | 2.49 |
| 0.73 | 13.12 | 9.36 | 2.44 |
| | 11.61 | 8.87 | 2.63 |
| | 12.32 | 8.58 | 2.42 |
| 1.29 | 12.55 | 7.81 | 2.90 |
| | 10.08 | 7.34 | 2.69 |

| | | | |
|---|---|---|---|
| | 11.94 | 7.33 | 2.74 |
| | 12.45 | 8.01 | 2.59 |
| | 11.76 | 8.02 | 2.79 |
| | 9.34 | 4.62 | 2.45 |
| | 8.77 | 4.03 | 2.43 |
| 1.92 | 8.45 | 4.81 | 2.51 |
| | 8.45 | 4.905 | 2.51 |
| | 8.75 | 5.01 | 2.51 |
| | 7.13 | 3.39 | 2.19 |
| | 7.09 | 3.35 | 2.15 |
| 3.88 | 8.39 | 3.59 | 2.21 |
| | 7.11 | 3.37 | 2.17 |
| | 7.43 | 3.39 | 2.26 |
| | 6.13 | 2.09 | 1.51 |
| | 6.23 | 2.49 | 1.29 |
| 10.93 | 6.63 | 2.69 | 1.68 |
| | 6.23 | 2.49 | 1.69 |
| | 6.73 | 3.19 | 1.79 |
| | 5.79 | 2.19 | 0.85 |
| | 5.57 | 1.59 | 1.07 |
| 21.84 | 6.17 | 2.19 | 1.19 |
| | 6.03 | 2.29 | 1.09 |
| | 5.43 | 1.66 | 1.15 |

## References


[1] S. Jeon, S. Son, S.Y. Lee, D. Chae, J.H. Bae, H. Lee, S.J. Oh, Multifunctional Daytime Radiative Cooling Devices with Simultaneous Light-Emitting and Radiative Cooling Functional Layers, ACS Applied Materials & Interfaces 12(49) (2020) 54763-54772.

[2] A.P. Raman, M.A. Anoma, L. Zhu, E. Rephaeli, S. Fan, Passive radiative cooling below ambient air temperature under direct sunlight, Nature 515(7528) (2014) 540-544.

[3] D. Lee, M. Go, S. Son, M. Kim, T. Badloe, H. Lee, J.K. Kim, J. Rho, Sub-ambient daytime radiative cooling by silica-coated porous anodic aluminum oxide, Nano Energy 79 (2021) 105426.

[4] S. Zeng, S. Pian, M. Su, Z. Wang, M. Wu, X. Liu, M. Chen, Y. Xiang, J. Wu, M. Zhang, Hierarchical-morphology metafabric for scalable passive daytime radiative cooling, Science 373(6555) (2021) 692-696.

[5] T. Li, Y. Zhai, S. He, W. Gan, Z. Wei, M. Heidarinejad, D. Dalgo, R. Mi, X. Zhao, J. Song, A radiative cooling structural material, Science 364(6442) (2019) 760-763.

[6] K. Panwar, M. Jassal, A.K. Agrawal, TiO2–SiO2 Janus particles treated cotton fabric for thermal regulation, Surface and Coatings Technology 309 (2017) 897-903.

[7] H. Zhang, J. Huang, D. Fan, Switchable radiative cooling from temperature-responsive thermal resistance modulation, ACS Applied Energy Materials 5(5) (2022) 6003-6010.

[8] Y. Song, Y. Li, B. Ge, J. Wang, J. Li, Self-Cleaning and Spectral Selective Membrane for Sustainable Radiative Cooling, ACS applied materials & interfaces (2023).

[9] Y. Chen, J. Mandal, W. Li, A. Smith-Washington, C.-C. Tsai, W. Huang, S. Shrestha, N. Yu, R.P.S. Han, A. Cao, Y. Yang, Colored and paintable bilayer coatings with high solar-infrared reflectance for efficient cooling, Science Advances 6(17) (2020) eaaz5413.

[10] T. Wang, Y. Wu, L. Shi, X. Hu, M. Chen, L. Wu, A structural polymer for highly efficient all-day passive radiative cooling, Nature Communications 12(1) (2021) 365.
